# Current use of percutaneous image-guided tumor ablation for the therapy of liver tumors: lessons learned from the registry of the German Society for Interventional Radiology and Minimally Invasive Therapy (DeGIR) 2018–2022

**DOI:** 10.1007/s00330-023-10412-w

**Published:** 2023-11-08

**Authors:** Sebastian Zensen, Arno Bücker, Mathias Meetschen, Johannes Haubold, Marcel Opitz, Jens M. Theysohn, Sara Schramm, Leonie Jochheim, Stefan Kasper, Michael Forsting, Benedikt Michael Schaarschmidt

**Affiliations:** 1 Institute of Diagnostic and Interventional Radiology and Neuroradiology, University Hospital Essen, Essen, Germany; 2 Department of Diagnostic and Interventional Radiology, University Hospital Homburg/Saar, Homburg, Germany; 3 Institute for Medical Informatics, Biometry, and Epidemiology, University Hospital Essen, Essen, Germany; 4 Department of Gastroenterology and Hepatology, University Hospital Essen, Essen, Germany; 5 West German Cancer Center, Department of Medical Oncology, University Hospital Essen, University of Duisburg-Essen, Essen, Germany

**Keywords:** Microwave ablation, Radiofrequency ablation, Hepatocellular carcinoma, Liver metastases

## Abstract

**Objectives:**

Percutaneous image-guided tumor ablation of liver malignancies has become an indispensable therapeutic procedure. The aim of this evaluation of the prospectively managed multinational registry of the voluntary German Society for Interventional Radiology and Minimally Invasive Therapy (DeGIR) was to analyze its use, technical success, and complications in clinical practice.

**Materials and methods:**

All liver tumor ablations from 2018 to 2022 were included. Technical success was defined as complete ablation of the tumor with an ablative margin.

**Results:**

A total of 7228 liver tumor ablations from 136 centers in Germany and Austria were analyzed. In total, 31.4% (2268/7228) of patients were female. Median age was 67 years (IQR 58–74 years). Microwave ablation (MWA) was performed in 65.1% (4703/7228), and radiofrequency ablation (RFA) in 32.7% (2361/7228). Of 5229 cases with reported tumor etiology, 60.3% (3152/5229) of ablations were performed for liver metastases and 37.3% (1950/5229) for hepatocellular carcinoma. The median lesion diameter was 19 mm (IQR 12–27 mm). In total, 91.8% (6636/7228) of ablations were technically successful. The rate of technically successful ablations was significantly higher in MWA (93.9%, 4417/4703) than in RFA (87.3%, 2061/2361) (*p* < 0.0001). The total complication rate was 3.0% (214/7228) and was significantly higher in MWA (4.0%, 189/4703) than in RFA (0.9%, 21/2361, *p* < 0.0001). Additional needle track ablation did not increase the rate of major complications significantly (24.8% (33/133) vs. 28.4% (23/81),* p* = 0.56)).

**Conclusion:**

MWA is the most frequent ablation method. Percutaneous image-guided liver tumor ablations have a high technical success rate, which is higher for MWA than RFA. The complication rate is generally low but is higher for MWA than RFA.

**Clinical relevance statement:**

Percutaneous image-guided liver ablation using microwave ablation and radiofrequency ablation are effective therapeutic procedures with low complication rates for the treatment of primary and secondary liver malignancies.

**Key Points:**

*• Percutaneous image-guided liver tumor ablations have a high technical success rate, which is higher for microwave ablation than radiofrequency ablation.*

*• Microwave ablation is the most frequent ablation method ahead of radiofrequency ablation.*

*• The complication rate is generally low but is higher for microwave ablation than radiofrequency ablation.*

## Introduction

Percutaneous image-guided tumor ablation is a minimally invasive interventional procedure that is routinely used for the treatment of malignant liver lesions [1]. Compared to surgical therapy, minimally invasive percutaneous ablations of primary and secondary liver tumors offer lower morbidity, mortality, and shorter hospital stays due to less invasiveness [2]. In addition, due to the parenchyma-sparing approach, local ablations can be performed repeatedly during the course of the disease and can be used in combination with surgical resection and systemic therapy [1]. In various guidelines, established indications for percutaneous image-guided ablation of liver tumors are early-stage hepatocellular carcinoma (HCC), intrahepatic cholangiocarcinoma (ICC), and liver metastases in colorectal carcinoma (CRC), but patients with hepatic oligometastatic malignancy of non-colorectal origin may also benefit from early ablation therapy [1, 3–10].

However, there is a continuous dispute regarding the different ways to conduct thermoablations in the liver due to a lack of prospective multicenter trials. A variety of ablation methods are available, with radiofrequency ablation (RFA) and microwave ablation (MWA) being the most commonly used [6]. For image guidance and needle navigation, CT is one of the most established techniques due to its wide availability and the possibility of visualizing the tumor in relation to adjacent structures for precise ablation planning [11]. However, ultrasound, and MRI are also available [9]. To overcome this lack of evidence, clinical registries might be important tools to provide clinical data and, consecutively, recommendations for the future use of thermal ablation in the liver.

DeGIR (Deutsche Gesellschaft für Interventionelle Radiologie und minimal-invasive Therapie) is the German Society for Interventional Radiology and Minimally Invasive Therapy and a member of the Cardiovascular and Interventional Radiological Society of Europe (CIRSE). A total of 303 centers from Germany, Austria, and Switzerland participate in the prospectively managed DeGIR registry, which is operated for quality assurance and research purposes.

The aim of this evaluation of the DeGIR registry was to analyze the use, technical success, and complications of percutaneous image-guided tumor ablations in the liver.

## Material and methods

In the present analysis, data sets on tumor ablations of the liver reported by all participating centers in the prospectively managed DeGIR registry from January 2018 until December 2022 were analyzed. Data were collected using an online data managing software (samedi GmbH). Participating centers submitted data online on a voluntary basis until the end of February for each previous year. The following mandatory parameters were analyzed: date of intervention, sex, age, inpatient or outpatient intervention, blood coagulation laboratory parameters (platelet count, international normalized ratio (INR), partial thromboplastin time (PTT)), use of antibiotics, liver segment, complications in the first 24 h, technical success. The following optional parameters were analyzed: repeated or multiple intervention, tumor etiology, lesion size, imaging modality, therapeutic intent, additive techniques, anesthesiological care, and ablation method. Laboratory parameters were reported as normal, pathological, or not determined. Technical success was defined as complete ablation of the tumor with an ablative margin beyond the limits of the tumor to achieve complete tumor destruction [1, 11]. Depending on the tumor etiology, the ablative margin was 0.5–1.0 cm [12, 13]. Complications were classified by severity into minor (A: no need for therapy, no consequences; B: symptomatic treatment and overnight observation, if necessary) and major (C: need for therapy, short hospitalization time (< 48 h); D: need for therapy, unplanned increase in treatment level, prolonged hospitalization time (> 48 h); E: permanent health damage; F: death) complications according to the SIR classification system for complications by outcome [14]. Ethical approval for this retrospective study was granted by the ethics committee of the University of Duisburg-Essen (22–10865-BO).

### Statistics and data analysis

Statistical analysis was performed using GraphPad Prism 5.01 (GraphPad Software). To determine normal distribution, D’Agostino-Pearson test was applied. Non-normally distributed data are reported as median and interquartile range (IQR). Non-normally distributed continuous variables were compared using Mann–Whitney and Kruskal–Wallis test. The chi-square test was used to compare complication and technical success rates by different parameters. *p* values lower than 0.05 were considered statistically significant. Due to the explorative nature of this study, no alpha error correction was performed.

## Results

### Patient characteristics

**Fig. 1:**
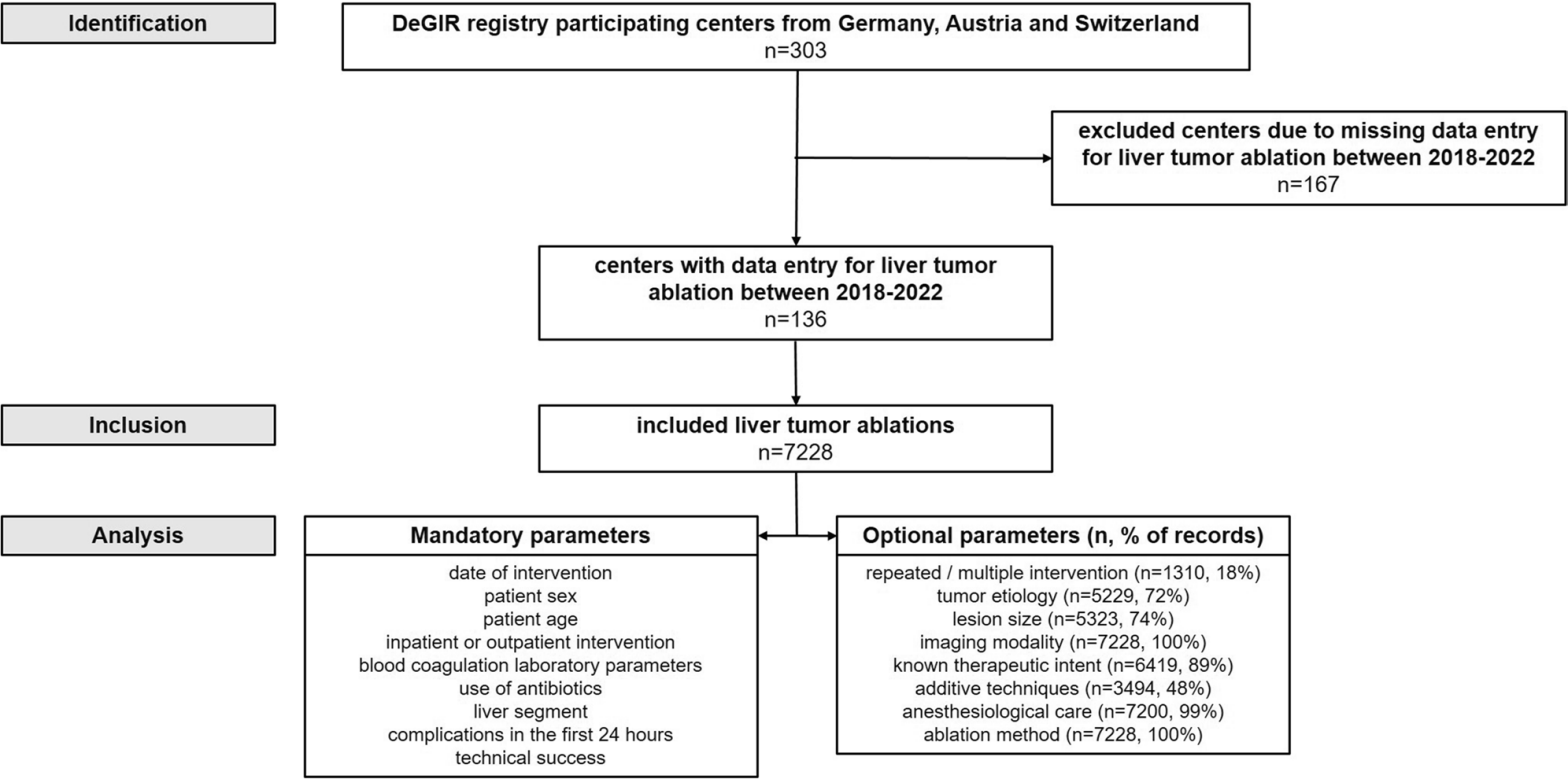
Study flow chart

From 2018 to 2022, a total of 7228 percutaneous liver tumor ablations from 136 centers in Germany and Austria were recorded in the DeGIR registry (Fig. 1). From 2018 to 2020, the number of ablations increased from 1163 to 1763 and decreased to 1436 in 2022 (Table 1). A total of 31.4% (2268/7228) of patients were female. Median age was 67 years (IQR 58–74 years, Table 2). Nearly all ablations (98.7%, 7134/7228) were performed as inpatient procedures. In total, 18.1% (1310/7228) of ablations were part of multiple or repeated interventions. The therapeutic goal was stated in 89% (6419/7228): of these, 70.0% (4491/6419) were performed in curative and 30.0% (1928/6419) in palliative intent.

**Table 1:** Frequency of percutaneous image-guided tumor ablations of the liver from 2018 to 2022

	2018	2019	2020	2021	2022	All years
Number of liver ablations (%)	1163 (16.1%)	1271 (17.6%)	1763 (24.4%)	1595 (22.1%)	1436 (19.9%)	7228 (100%)
Change in frequency to the previous year	N/A	+ 9.3%	+ 38.7%	-9.5%	-10.0%	
Intervention type						
MWA	705 (60.6%)	855 (67.3%)	1122 (63.6%)	1043 (65.4%)	978 (68.1%)	4703 (65.1%)
RFA	414 (35.6%)	392 (30.8%)	603 (34.2%)	531(33.3%)	421 (29.3%)	2361 (32.7%)
IRE	36 (3.1%)	16 (1.3%)	8 (0.5%)	8 (0.5%)	9 (0.6%)	77 (1.1%)
ECT	0 (0%)	0 (0%)	20 (1.1%)	2 (0.1%)	4 (0.3%)	26 (0.3%)
LITT	2 (0.2%)	0 (0%)	0 (0%)	1 (0.1%)	0 (0%)	3 (0.1%)
not defined	5 (0.4%)	7 (0.6%)	4 (0.2%)	5 (0.3%)	22 (1.5%)	43 (0.5%)
Multiple ablation procedures used	1 (0.1%) (1 RFA + MWA)	1 (0.1%) (1 RFA + MWA)	6 (0.3%) (2 RFA + MWA, 1 MWA + IRE, 3 MWA + ECT)	5 (0.3%) (3 RFA + MWA, 1 RFA + LITT, 1 MWA + IRE)	2 (0.1%) (1 RFA + MWA, 1 MWA + ECT)	15 (0.2%)

*MWA*, microwave ablation; *RFA*, radiofrequency ablation; *IRE*, Irreversible electroporation; *ECT*, electrochemotherapy; *LITT*, laser-induced thermotherapy

**Table 2:** Overview of baseline patient and tumor characteristics differentiated by microwave ablations and radiofrequency ablations

		Ablation technique	
Characteristics	Total	Microwave ablation (MWA)	Radiofrequency ablation (RFA)
Number of patients	7228 (100%)	4703 (65.1%)	2361 (32.7%)
Gender (female)	2268 (31.4%)	1505 (32.0%)	708 (30.0%)
Age (years)	67 (IQR 58–74)	67 (IQR 58–75)	66 (IQR 57–74)
Reported tumor etiology	5229	3617	1528
Metastases	3152 (60.3%)	2153 (59.5%)	966 (63.2%)
Hepatocellular carcinoma (HCC)	1950 (37.3%)	1384 (38.3%)	521 (34.1%)
Intrahepatic cholangiocarcinoma (ICC)	105 (2.0%)	77 (2.1%)	24 (1.6%)
Benign primary tumor	22 (0.4%)	3 (0.1%)	17 (1.1%)
Lesion size	19 mm (IQR 12–27 mm)	20 mm (IQR 12–26 mm)	17 mm (10–27 mm)
Ablation of the needle track	2910 (40.1%)	2505 (53.3%)	364 (15.4%)
Technical success rate	6636 (91.8%)	4417 (93.9%)	2061 (87.3%)
Complication rate	214 (3.0%)	189 (4.0%)	21 (0.9%)

Parameters are given as *n* (%) or median values with interquartile range (IQR)

### Etiology and characteristics of ablated liver lesions

Tumor etiology was reported in 72.3% (5229/7228, Table 2). Of these, metastases accounted for 60.3% (3152/5229) and HCC for 37.3% (1950/5229). 2.0% (105/5229) of ablations were performed for ICC and 0.4% (22/5229) for a benign primary tumor. The proportion of curatively intended ablations was slightly higher for HCC (71.0%, 1385/1950) than for metastases (67.1%, 2115/3152) and ICC (62.9%, 66/105, *p* = 0.006). Lesion size was reported in 74% (5323/7228) of all ablations, with a median lesion diameter of 19 mm (IQR 12–27 mm). Among the different ablated tumor entities, metastases (median 17 mm, IQR 10–25 mm) were statistically significantly smaller than HCCs (median 20 mm, IQR 13–28 mm, *p* < 0.0001). In total, 36.7% (2656/7228) of lesions were located in the left and 63.3% (4572/7228) in the right liver lobe. The lesion distribution per segment is depicted in Table 3.

**Table 3:** Percutaneous image-guided ablations of the liver differentiated by lobe and segment

Liver lobe	Segment	*n*	%
Left liver lobe		2656	36.7
	I	638	8.8
	II + III	779	10.8
	IVa	877	12.1
	IVb	362	5.0
Right liver lobe		4572	63.3
	V	955	13.2
	VI	1000	13.9
	VII	1222	16.9
	VIII	1395	19.3

### Percutaneous image-guided liver ablation methods, modality for image guidance, and periprocedural specifications

**Fig. 2:**
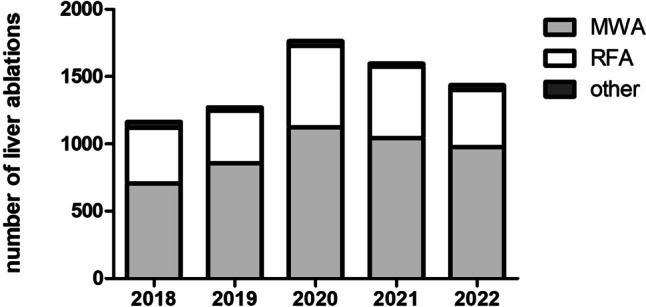
Frequency of percutaneous image-guided tumor ablations of the liver from 2018 to 2022. MWA = microwave ablation; RFA = radiofrequency ablation

For image guidance, CT was used in 92.2% (6664/7228), MRI in 2.3% (164/7228), and ultrasound in 4.1% (294/7228), while a combined approach (e.g., ultrasound and CT) was performed in 1.5% (106/7228). The main ablation technique was MWA in 65.1% (4703/7228) and RFA accounted for 32.7% (2361/7228). With proportions of at least 60.6% in 2018 and at most 68.1% in 2022, MWA was consistently the most frequently used method for liver ablation (Fig. 2, Table 1). Liver tumors treated with MWA were larger than liver tumors ablated using RFA (MWA: median tumor size 20 mm, IQR 12–26 mm, RFA: median tumor size 17 mm, IQR 10–27 mm, *p* < 0.0001, Table 2). Further ablation methods (irreversible electroporation, electrochemotherapy, laser-induced thermotherapy) and combined methods accounted for only 2.2% (161/7228). Additive techniques included ablation of the needle track in 40.3% (2910/7228), protective cooling of adjacent tissue in 7.2% (520/7228), and protective organ relocation in 0.9% (64/7228). Ablation of the needle track was performed with equal frequency in the right and left liver lobe (right liver lobe: 40.3%, 1842/4572; left liver lobe: 40.2%, 1067/2656, *p* = 0.92). However, this additional technique was reported significantly more often in patients treated with MWA than RFA (MWA: 53.3%, 2505/4703; RFA: 15.4%, 364/2361, *p* < 0.0001, Table 2). Preinterventional antibiotics were administered in less than half of all ablations (47%, 3394/7228). 82.9% (5992/7228) of ablations were performed under general anesthesia, while analgosedation was performed in 10.8% (780/7228) and local anesthesia in 5.9% (428/7228). Reported laboratory parameters for blood coagulation are shown in Table 4. In the majority of patients, all reported coagulation parameters were normal (78.7%, 5685/7228).

**Table 4:** Frequency of complications of percutaneous image-guided tumor ablations of the liver in relation to coagulation-related laboratory parameters

			No complication		Complication		
Laboratory parameter		*n* (%)	*n*	%	*n*	%	*p* value
Platelet count	Normal	5880 (81.4%)	5705	97.02	175	2.98	0.301
	Pathological	837 (11.6%)	806	96.30	31	3.70	
	Not reported	511 (7.0%)					
International Normalized Ratio (INR)	Normal	6359 (88.0%)	6166	96.96	193	3.04	0.786
	Pathological	380 (5.3%)	367	96.58	13	3.42	
	Not reported	489 (6.7%)					
Partial thromboplastin time (PTT)	Normal	6508 (90.0%)	6310	96.96	198	3.04	0.992
	Pathological	214 (3.0%)	207	96.73	7	3.27	
	Not reported	506 (7.0%)					

### Technical success of percutaneous image-guided tumor ablations in the liver

**Fig. 3:**
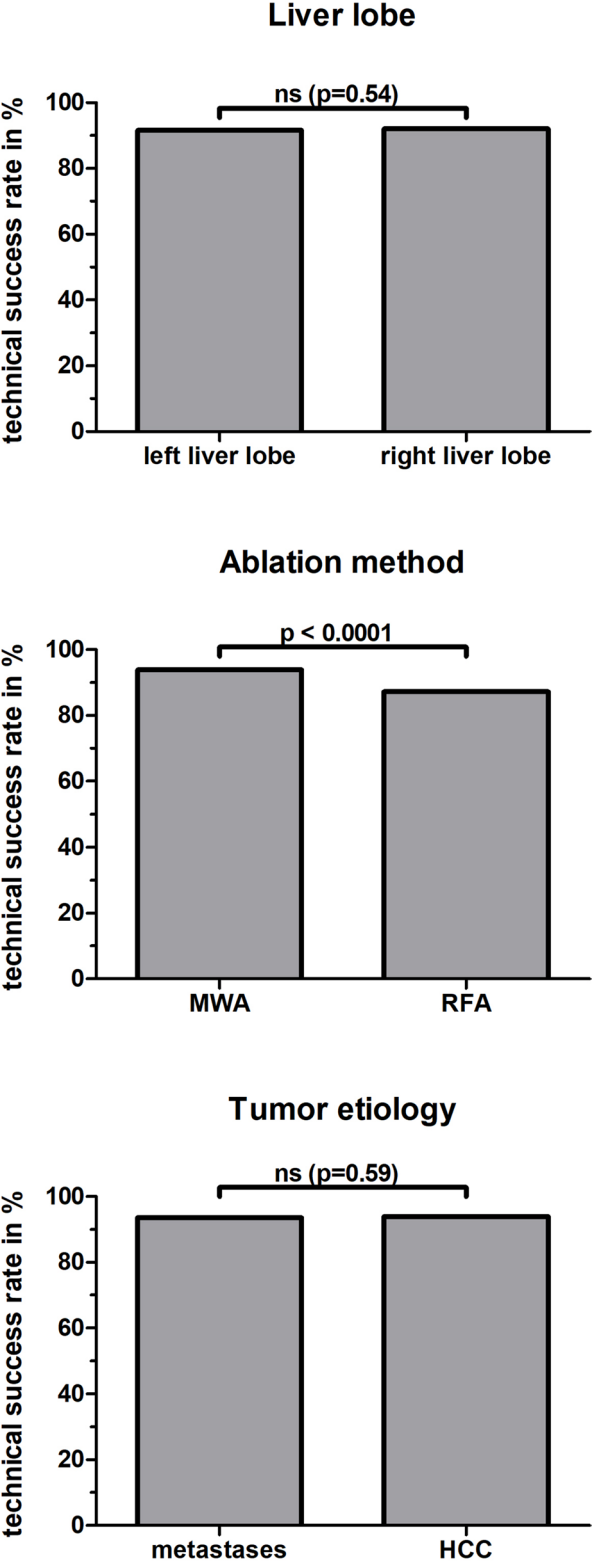
Technical success rates in percutaneous image-guided tumor ablation of the liver differentiated by liver lobe, ablation method, and tumor etiology. MWA = microwave ablation; RFA = radiofrequency ablation; HCC: hepatocellular carcinoma. ns = not significant (*p* > 0.05)

Tumor ablation was technically successful in 91.8% (6636/7228), while in 8.2% (592/7228) the lesion could only be ablated incompletely or without an ablative margin. The technical success rate was slightly higher in ablations with curative (93.2%, 4185/4491) than with palliative intent (90.5%, 1745/1928, *p* = 0.0002). The rate of technically successful ablations was comparable in both liver lobes (right liver lobe: 92.0%, 4205/4572; left liver lobe: 91.5%, 2431/2656, *p* = 0.54). The rate of technically successful ablations was significantly higher with MWA (93.9%, 4417/4703) than with RFA (87.3%, 2061/2361, *p* < 0.0001) (Fig. 3, Table 2). The technical success rate was comparable for the ablation of metastases (93.5%,2948/3152) and HCC (93.9%, 1832/1950, *p* = 0.59).

### Complications of percutaneous image-guided tumor ablations in the liver

The total complication rate was 3.0% (214/7228). About a quarter of complications (26.2%, 56/214) were major complications according to the SIR classification (category C to F). There were no major complications in categories E (permanent health damage) or F (death). Among the reported major complications, in category D (need for therapy, unplanned increase in treatment level, prolonged hospitalization > 48 h) arterial bleedings accounted for 35% (8/23), while most major complications in category C (need for therapy, short hospitalization time < 48 h) were pulmonary complications (61%, 20/33, Table 5). Among minor complications, in category B (symptomatic treatment and overnight observation, if necessary), pulmonary complications were most common at 31% (23/76), and venous bleeding at 25% (19/76). Among the minor complications of the lowest category A (no need for therapy, no consequences), parenchymal bleeding with 29% (24/82) and venous bleeding with 23% (19/82) were the most frequent.

**Table 5:** Complications of percutaneous image-guided tumor ablations of the liver differentiated by complication type

		Classification of complication*					
		Minor		Major			
Type of complication	*n* (%)	A	B	C	D	E	F
Arterial bleeding	29 (13.6%)	3	12	6	8	0	0
Cardiac complication	1 (0.5%)	0	0	0	1	0	0
Material malposition	1 (0.5%)	0	1	0	0	0	0
Drug side effect	1 (0.5%)	0	1	0	0	0	0
Organ dysfunction	1 (0.5%)	0	0	0	1	0	0
Parenchymal bleeding	47 (22.0%)	24	14	4	5	0	0
Parenchymal infarction	1 (0.5%)	1	0	0	0	0	0
Pulmonary complication	66 (30.8%)	19	23	20	4	0	0
Venous bleeding	43 (20.1%)	18	19	3	3	0	0
Venous occlusion	1 (0.5%)	1	0	0	0	0	0
Others	23 (10.7%)	16	6	0	1	0	0
All complications	214 (100%)	82 (38.3%)	76 (35.5%)	33 (15.4%)	23 (10.8%)	0 (0%)	0 (0%)

^*^according to the SIR Classification System for Complications by Outcome [14]

Complication rates of tumor ablation were comparable in both liver lobes (right liver lobe: 3.2%, 147/4572; left liver lobe: 2.5%, 67/2656, *p* = 0.11). The complication rate was significantly higher in MWA with 4.0% (189/4703) than in RFA with 0.9% (21/2361, *p* < 0.0001, Table 2). Likewise, major complications were significantly more frequent in MWA at 1.0% (47/4703) than in RFA at 0.3% (8/2361, *p* = 0.005). Complication rates according to the etiology of the ablated tumor were not statistically significantly different: metastases 3.6% (115/3152), HCC 2.6% (50/1950), and ICC 2.9% (3/105) (*p* = 0.101).

Interventions with additional ablation of the needle track had a significantly higher complication rate of 4.6% (133/2910) compared to 1.9% (81/4318) without ablation of the needle track (*p* < 0.0001). However, the proportion of major complications (category C to F) did not differ significantly (major complications after ablation of the needle track; 24.8%, 33/133; major complications without additional ablation of the needle track: 28.4%, 23/81, *p* = 0.56). The proportion of patients with complications is slightly higher in the case of pathological coagulation parameters, but the impact of pathological coagulation parameters on the occurrence of complications was not significant (pathological vs. normal: platelet count: 3.70% vs. 2.98%, *p* = 0.301; INR: 3.42% vs. 3.04%, *p* = 0.786; PTT: 3.27% vs. 3.04%, *p* = 0.992, Table 4).

## Discussion

Percutaneous image-guided tumor ablations for the therapy of focal liver lesions have become an indispensable therapeutic procedure for the treatment of liver tumors. Despite the high clinical relevance of percutaneous tumor ablation, further research is mandatory to increase its clinical use and improve therapeutic success rates even further. This analysis of the multinational DeGIR registry yields four key findings. First, our study shows that percutaneous image-guided thermal ablation of the liver has a high technical success rate with a low complication rate, for both curative and palliative intent. Second, MWA was the preferred ablation compared to RFA by the registry participants and had a higher technical success rate. Third, the complication rate is higher in MWA than in RFA. Fourth, needle track ablation, a technique necessary to circumvent manual tumor cell dislocation, does not increase the number of major complications.

In the last years, percutaneous ablation has evolved into an important therapeutic option for primary and secondary liver malignancies due to its minimal invasiveness and the possibility of preserving non-tumorous liver tissue by precise image-guided needle placement. Hence, it is not only an important treatment option for small liver tumors but can also be combined with systemic, intra-arterial, or surgical therapies, and is an indispensable modality to treat recurrent tumors in a palliative setting [1]. Hence, this treatment is recommended by several guidelines. In the treatment of HCC, percutaneous thermal ablation by RFA or MWA is a treatment option in BCLC (Barcelona Clinic Liver Cancer) stage 0 (single nodule < 2 cm, preserved liver function) and stage A (2–3 nodules, < 3 cm) tumors with preserved liver function [4–7, 9]. For HCC up to 3 cm, resection and ablation are equivalent, whereas ablation should be considered especially in case of unfavorable localization for resection, and also depending on further patient factors (liver function, extrahepatic health status) [4, 5, 9]. In ICC, the current ESMO (European Society for Medical Oncology) guideline recommends ablation for the treatment of tumors < 3 cm in patients with contraindications for surgery [15].

The majority of ablated HCCs in our study were small (median 20 mm, 75^th^ percentile 28 mm), well fitting the guidelines that predominantly small HCCs less than 3 cm should undergo ablation [3–5, 9]. Our study shows that these guidelines for HCC with indications including tumor size are widely accepted and used in Germany and Austria. However, the major proportion of ablations in our study was performed for liver metastases, which is specifically recommended for the treatment of CRC liver metastases by the ESMO but may also be indicated for liver metastases from other malignancies [8]. Furthermore, ablation can also achieve a comparable outcome to resection in liver metastases, which was shown for overall survival for patients with singular CRC liver metastases smaller than 3 cm [3, 8]. In the ESMO guideline for CRC liver metastases, RFA plays a role in lesion size up to a maximum of 2–3 cm, especially in recurrence but also in combination with surgical procedures [8]. In addition, the prospective randomized phase 2 CLOCC trial has shown that ablation prolongs overall survival in unresectable CRC liver metastases [16]. In addition, the prospective randomized phase 3 COLLISION trial analyzes whether ablation of CRC liver metastases up to 3 cm is non-inferior and therefore should be preferred over resection due to lower morbidity and mortality and cost reduction [17]. The sizes of the ablated metastases (median 17 mm, 75^th^ percentile 25 mm) in our study show a wide application of these size thresholds, although unfortunately, no information on the primary tumor was available in the registry. In addition, percutaneous tumor ablation plays an important role as a bridging therapy for patients with HCC within Milan criteria prior to liver transplantation to prevent dropout from the waiting list [18]. In addition, our results show that liver ablations play an important role also in local therapy in palliative setting with 30% of ablations performed with palliative intent, although data and recommendations on this remains scarce in the literature and guidelines.

We demonstrated that almost exclusively hyperthermic ablations were used for the treatment of liver tumors. Both MWA and RFA induce heat-based thermal cytotoxicity, in RFA by electric current in the RF range, and in MWA by electromagnetic energy [4, 11]. Tumor necrosis due to thermal cytotoxicity is affected by heat convection in thermal ablations, which is particularly worsened by blood flow in adjacent vessels. MWA is less susceptible to this so-called heat sink effect, which is an advantage compared to RFA [19, 20]. In principle, technical success with a completely ablated tumor with an ablative margin is a strong predictor of complete tumor eradication [8, 20, 21]. In technically successful RFA, but also in RFA followed by resection in cases with initial local failure, survival is likely to be almost identical compared with resection [4, 22]. Overall, the rate of technically successful ablations in our study was very high, both for curative and palliative intended ablations, in both liver lobes and both for liver metastases and HCC. However, in contrast to other studies, the rate of technically successful ablations in our study was significantly higher for MWA than for RFA [23]. The results of studies comparing the therapeutic effects of MWA and RFA have been variable and inconclusive [24–27]. In addition to RFA, MWA has emerged as an effective ablation method, as comparable therapeutic efficacy and complication rates have been demonstrated in the treatment of HCC and liver metastases in CRC [28, 29]. MWA has advantages in ablating larger tumors, shorter treatment time, and easier placement of an antenna to achieve a comparable ablation zone [23, 26]. In the past, in various guidelines, only RFA was recommended as the standard ablation method, but on the basis of stronger evidence with comparable results, MWA has been included as an equivalent ablation method in the German S3 guideline for HCC as well as the guideline for HCC of the Asian Pacific Association for the Study of the Liver (APASL) [6, 9]. In our study, about two-thirds of liver ablations were performed using MWA and about one-third using RFA. Our results show the important role of MWA as the most frequently performed ablation method with a higher rate of technical success in Germany and Austria.

Image-guided ablation of focal liver lesions can result in puncture- and thermal-related complications [1]. With 3.0%, the overall complication rate was low and comparable to previous studies, with major complications accounting for only 0.8% [30, 31]. In our study, it is remarkable that the complication rate was more than four times higher in MWA than in RFA. In contrast to the literature, our study also showed statistically significantly more frequent major complications in MWA compared with RFA [23]. Furthermore, our results show that additive ablation of the needle track more than doubled the complication rate. Percutaneous ablations have a low risk of needle track seeding with reported rates of 0.6–1.7%, which in the worst case, e.g., due to peritoneal carcinomatosis, can dramatically worsen the patient's prognosis and thwart further curative treatment approaches [32, 33]. Although the overall complication rate was increased by needle track ablation, predominantly minor complications were observed. Therefore, our results support the CIRSE recommendation as the benefit continues to outweigh the potential risks of this additive procedure [1].

Limitations of this study include that, because of the voluntary character of the registry, not all information is complete which may result in significant reporting bias, in particular for optional parameters. This is especially true for additional procedures such as track ablation, which were only reported in 48%. However, for many important information, an entry has been mandatory. In principle, all participating centers are encouraged to enter all interventions into the registry. Furthermore, tumor size and complication evaluation were assessed by the participating centers themselves and thus cannot be independently validated. Therefore, the representativeness of the data cannot be proven with certainty. Nevertheless, although the registry is managed without monitoring and lacks information on tumor response and long-term outcomes, it provides a unique overview of the use of thermal ablation of the liver in a large patient cohort.

## Conclusion

Percutaneous image-guided tumor ablations for the therapy of focal liver lesions have a high technical success rate and a low complication rate, for both curative and palliative intent and for both HCC and metastases. In Germany and Austria, MWA is the preferred ablation method before RFA and has a higher technical success rate.

## Abbreviations

APASL: Asian Pacific Association for the Study of the Liver
BCLC: Barcelona Clinic Liver Cancer
CIRSE: Cardiovascular and Interventional Radiological Society of Europe
CRC: Colorectal carcinoma
DeGIR: Deutsche Gesellschaft für Interventionelle Radiologie und minimal-invasive Therapie
ESMO: European Society for Medical Oncology
HCC: Hepatocellular carcinoma
ICC: Intrahepatic cholangiocarcinoma
INR: International normalized ratio
IQR: Interquartile range
MWA: Microwave ablation
PTT: Partial thromboplastin time
RFA: Radiofrequency ablation

## Declarations

### Guarantor

The scientific guarantor of this publication is Sebastian Zensen.

### Statistics and biometry

No complex statistical methods were necessary for this paper.

### Study subjects or cohorts overlap

No study subjects or cohorts have been previously reported.

### Methodology

• retrospective

• observational

• multicenter study
